# Loss of PTEN in Fallopian Tube Epithelium Results in Multicellular Tumor Spheroid Formation and Metastasis to the Ovary

**DOI:** 10.3390/cancers11060884

**Published:** 2019-06-25

**Authors:** Matthew Dean, Vivian Jin, Tova M. Bergsten, Julia R. Austin, Daniel D. Lantvit, Angela Russo, Joanna E. Burdette

**Affiliations:** 1 Department of Pharmaceutical Sciences, Center for Biomolecular Science, University of Illinois at Chicago, Chicago, IL 60607, USA; mjdean@illinois.edu (M.D.); vivian.ww.jin@gmail.com (V.J.); bergste1@uic.edu (T.M.B.); jausti6@uic.edu (J.R.A.); lantvit@uic.edu (D.D.L.); russoa@uic.edu (A.R.); 2 Department of Animal Science, University of Illinois at Urbana-Champaign, Urbana, IL 61801, USA

**Keywords:** ovary, cancer, PTEN, metastasis, oviduct, fallopian tube, spheroid

## Abstract

High-grade serous ovarian cancer (HGSOC) can originate in the fallopian tube and then spread to the ovary. Our objective was to evaluate the role of multicellular tumor spheroids (MTS) in ovarian metastasis. By testing a panel of murine oviductal epithelial (MOE) cells with genetic alterations mimicking those seen in HGSOC, we found that loss of PTEN allowed MTS formation under ultra-low adhesion conditions. Confirming these results in vivo, MTS-like structures were observed in the oviducts of PAX8^Cre/+^ PTEN^flox/flox^ mice. MOE PTEN^shRNA^ cells could incorporate up to 25% wild type cells into MTS, while higher percentages of wild type cells resulted in a loss of MTS formation. MTS formation allowed MOE PTEN^shRNA^ cells to survive better under ultra-low adhesion conditions than control cells. MTS also attached to the ovarian stroma, as would be exposed during ovulation. Interestingly, MTS more robustly cleared monolayers of murine ovarian surface epithelia than murine ovarian fibroblasts. When xenografted into the ovarian bursa, OVCAR8 MTS were able to form tumors in the ovary at a similar rate as an equal number of OVCAR8 cells grown on traditional cell culture plastic. In conclusion, loss of a single gene (PTEN) allows the fallopian tube epithelia to form MTS, which survive better under ultra-low adhesion conditions, attach to the extracellular matrix exposed during ovulation, and colonize the ovary. These results suggest that MTS may contribute to seeding of the ovary in HGSOC patients.

## 1. Introduction

The identification of precursor lesions in the fallopian tube epithelia (FTE) has led to the hypothesis that high-grade serous ovarian cancer (HGSOC) originates in the fallopian tube [1]. The FTE origin of HGSOC is now supported by genomic, transcriptomic, and proteomic studies indicating that HGSOC is more similar to the FTE than the ovarian surface epithelium (OSE) [2,3,4], and retrospective studies showing that tubal ligation and salpingectomy are associated with a reduction in a woman’s risk for ovarian cancer [5,6]. Finally, multiple mouse models have been developed in which transformed FTE cells colonize the ovary [7,8,9].

In the current model of HGSOC development, mutations in the p53 gene lead to protein stabilization in the FTE, which is termed a p53 signature by pathologists. Eventually, other tumor suppressors are lost or oncogenes amplified (e.g., PTEN and KRAS, respectively), resulting in a serous tubal intraepithelial carcinoma (STIC), which subsequently metastasizes to the ovary and peritoneum [3,10]. The reasons STICs metastasize to the ovary are not completely clear, but chemotactic factors have been implicated in this early step of spread [11,12,13]. Tumorigenic cells likely adhere to the extracellular matrix (ECM) exposed during ovulation [12,14]. However, previous studies on this subject have assumed that individual cells mediate colonization of the ovary; the possibility that aggregates of tumor cells from the fallopian tube colonize the ovary remains relatively unexplored.

Multicellular tumor spheroids (MTS) are structures formed when multiple cells are placed under ultra-low adhesion (ULA) or three-dimensional (3D) conditions and form a compact, spherical structure [15]. Typically, MTS are thought to better model the tumor microenvironment than cells grown as a monolayer, due to their 3D structure, hypoxic center, and increased chemoresistance [15,16]. MTS-like structures are present in ascites from HGSOC patients, leading many investigators to suggest that they mediate metastasis from the ovary to the peritoneal organs [17,18,19]. A recent study using mCherry- and green fluorescent protein (GFP)-tagged ovarian cancer cells has suggested that MTS-like structures in ascites form in response to multi-cell clusters detaching from the ovarian tumors rather than the aggregation of individual cells in ascites or clonal expansion of individual cells after exfoliation [20]. Multiple studies have now observed MTS structures in the fallopian tubes of cancer patients [21,22]. Combined with the new knowledge that the majority of HGSOC cases originate in the fallopian tube, we hypothesize that MTS could mediate metastasis from the fallopian tube to the ovary.

When cultured under ULA conditions, cancer cell lines from the same type of cancer differ in their ability to form MTS structures [23], suggesting that mutations and differing gene expression in cancer cell lines impact their ability to aggregate in 3D. The objectives of the current project were to (1) identify altered genes or pathways that enhance the ability of FTE cells to form MTS and (2) explore the ability of FTE-derived MTS to colonize the ovary.

## 2. Results

### 2.1. Loss of PTEN Induces MTS Formation in Vitro and in Vivo

**Figure 1:**
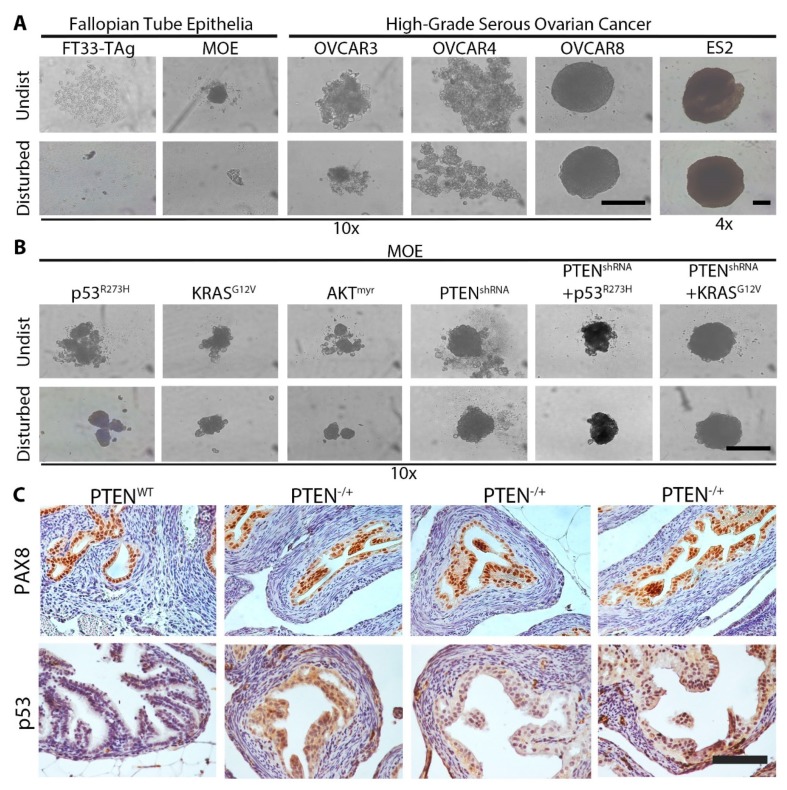
Loss of PTEN induces multi-cellular tumor spheroid (MTS) formation in vitro and in vivo. (**A**,**B**) Representative images of structures formed at seven days of culture in ultra-low adhesion (ULA) plates. Images were taken from undisturbed cells (undist) or after pipetting the contents of each well up and down (disturbed). Scale bar = 250 μm. (**C**) Immunohistochemistry for PAX8 and p53 in the fallopian tubes of PAX^Cre/+^ PTEN^+/+^ (PTEN^WT^) or PAX8^Cre/+^ PTEN^flox/+^ (PTEN^−/+^) mice. Scale bar = 100 μm. *n* = 4–6. Legend: MOE, murine oviductal epithelial.

First, we investigated whether normal FTE or ovarian cancer cell lines could form MTS structures. To accomplish this goal, we plated 500 FT33-TAg (human FTE-derived line) cells, murine oviductal epithelial (MOE) cells, and ovarian cancer cells (OVCAR3, OVCAR4, OVCAR8, and ES2) into ULA plates with a round bottom and incubated these for seven days. Structures in each well were imaged before and after being disturbed by pipetting to confirm the presence of aggregates. Neither cell line representing normal FTE cells (MOE and FT33-TAg) were able to form any type of 3D structure. Most OVCAR3 cells remained unattached to one another with only very small structures forming, while OVCAR4 cells formed large, irregularly shaped structures. By contrast, OVCAR8 formed spherical MTS with a smooth appearance, which is consistent with previous reports [23]. ES2 cells also formed spherical, smooth MTS. ES2 MTS were 73% bigger than those formed by OVCAR8 cells (Figure 1A and Figure S1A; *p* < 0.0001).

To determine which pathways in the FTE could drive MTS formation, we tested a panel of transgenic MOE cell lines engineered to mimic pathway alterations commonly observed in HGSOC patients: mutation in p53 (p53^R273H^), amplification of KRAS (KRAS^G12V^), activation of AKT signaling (AKT^myr^), and loss of PTEN protein (PTEN^shRNA^). We also tested these alterations in various combinations (PTEN^shRNA^ + p53^R273H^ and PTEN^shRNA^ + KRAS^G12V^) [7,24]. p53^R273H^, KRAS^G12V^, AKT^myr^, and KRAS^G12V^ MOE cells formed small, irregularly shaped structures. However, MOE cells expressing PTEN^shRNA^ formed typical MTS structures (Figure 1B). p53^R273H^ combined with PTEN^shRNA^ resulted in a 24% reduction in MTS size (*p* < 0.01), while MOE PTEN^shRNA^ + KRAS^G12V^ MTS tended to be slightly larger than MOE PTEN^shRNA^ MTS (*p* = 0.067; Figure 1B and Figure S1B). Control cells (MOE NEO and MOE SCR^shRNA^) did not form structures under ULA conditions (Figure S1C), similarly to wild type MOE cells.

To confirm these results in vivo, we next analyzed the fallopian tubes (or oviducts) of PAX8^Cre/+^ PTEN^+/+^ (hereafter referred to as FTE PTEN^WT^ mice) and PAX^cre/+^ PTEN^flox/+^ (hereafter referred to FTE PTEN^−/+^ mice) via immunohistochemistry. The fallopian tube of FTE PTEN^WT^ mice had a normal architecture which was positive for PAX8 and negative for p53. The fallopian tube lumen of FTE PTEN^WT^ mice (*n* = 3) was devoid of any MTS-like structures (Figure 1C). By contrast, FTE PTEN^−/+^ mice displayed hyperplasia in the FTE that stained positive for PAX8 and p53. Numerous areas of the tubal epithelium exhibited intense hyperplasia. Multi-cellular structures were present in the lumen of the oviducts, similar to MTS, and appeared to be detaching from the FTE. Some of the MTS appeared completely detached from the FTE inside the lumen of the fallopian tube (Figure 1C). Hyperplasia in the fallopian tubes of PAX^cre/+^ PTEN^flox/flox^ mice filled the lumen, precluding analysis of MTS-like structures.

**Figure 2:**
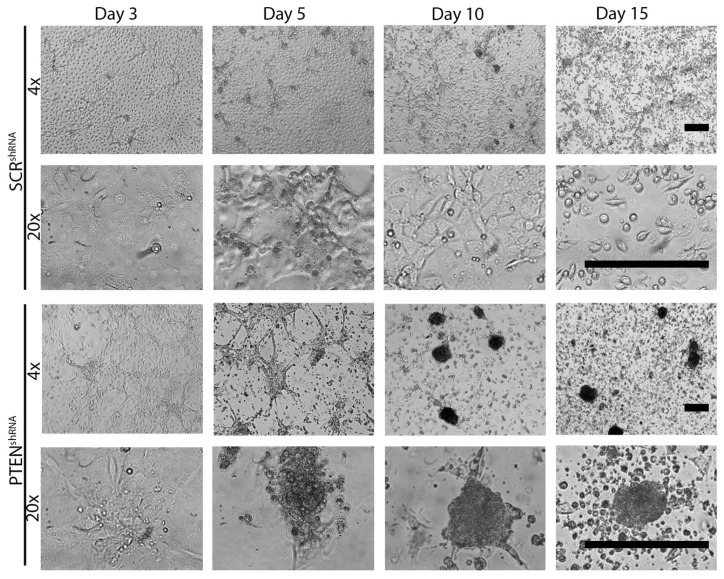
Overgrowth of fallopian tube epithelia (FTE) cells lacking PTEN results in budding of MTS-like structures. Representative images of MOE SCR^shRNA^ (top) and MOE PTEN^shRNA^ (bottom) of monolayers and structures formed over 15 days of growth. Scale bar = 250 μm. *n* = 4.

To confirm that MOE PTEN^shRNA^ cells were able to form MTS better than MOE SCR^shRNA^ cells, we examined MTS formation in a budding model [25]. Cells (25,000) were plated in 24-well plates and allowed to overgrow. By Day 3, both cell lines had formed a near confluent monolayer. MOE SCR^shRNA^ formed a few small MTS-like structures by Day 10. By Day 15, most MOE SCR^shRNA^ had detached and were floating as single cells in the media. The surviving MOE SCR^shRNA^ cells formed a monolayer with very few MTS-like structures. By contrast, by Day 5 MOE PTEN^shRNA^ cells had started to grow on top of each other. By Days 10–20, clear MTS-like structures had formed, with most structures then detaching from the monolayer underneath (Figure 2). The MTS-like structures formed by MOE PTEN^shRNA^ cells were ~9-fold more abundant and 88% larger than the structures formed by SCR^shRNA^ cells (Figure S2A,B; *p* < 0.05). These results suggest that loss of PTEN is a driver of MTS formation in the FTE both in vitro and in vivo.

### 2.2. Wild Type Cells Can Be Incorporated into MTS in Small Numbers

**Figure 3:**
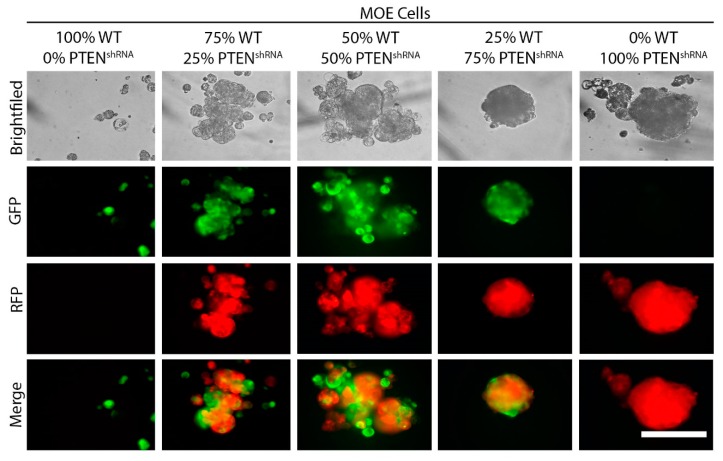
MTS formed by MOE PTEN^shRNA^ cells are able to incorporate some wild type (WT) MOE cells, but too many wild type cells prevents MTS formation. Representative images of structures formed with MOE wild type cells tagged with green fluorescent protein (GFP) and MOE PTEN^shRNA^ cells tagged with red fluorescent protein (RFP) were mixed at indicated ratios and cultured under ULA conditions for seven days. *n* = 3. Scale bar = 250 μm.

We sought to determine if non-tumorigenic cells expressing PTEN could be incorporated into the MTS formed by MOE PTEN^shRNA^ cells. To this end, we mixed wild type MOE cells tagged with GFP and MOE PTEN^shRNA^ cells tagged with red fluorescent protein (RFP) in 0/100%, 25/75%, 50/50%, 75/25%, and 100/0% ratios and plated 500 total cells under ULA conditions for seven days. As expected, 100% MOE PTEN^shRNA^ RFP cells formed MTS. Even when 25% of the cells were wild type, typical MTS structures formed. Fluorescence microscopy showed that the MOE GFP cells were incorporated into the MTS, being interspersed among the MOE PTEN^shRNA^ RFP cells. However, when the percentage of wild type MOE cells was increased to 50% or 75%, the ability to form spherical MTS was lost. The RFP and GFP positive cells still aggregated together but in smaller, irregularly shaped structures. At 100% MOE GFP cells, very small or no structures formed (Figure 3). This suggests that MTS formed in vivo might contain a small percentage of normal cells interspersed with tumor cells, but that a high enough concentration of non-tumorigenic cells can disrupt this process.

### 2.3. Loss of PTEN Increases Cell Survival under ULA Conditions

**Figure 4:**
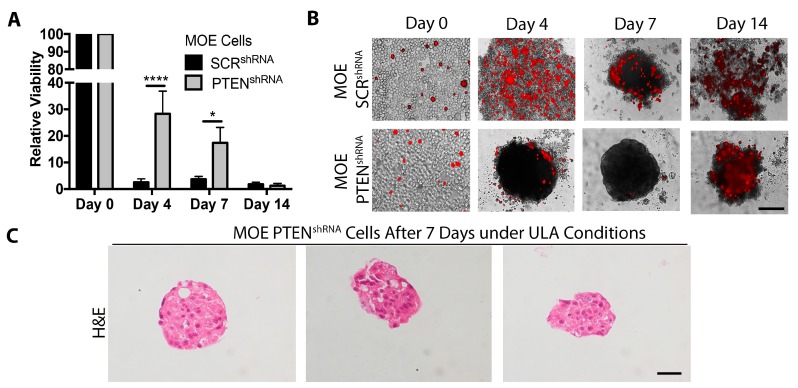
MOE PTEN^shRNA^ MTS survive better under ULA conditions. (**A**) Relative viability of MOE SCR^shRNA^ and MOE PTEN^shRNA^ cells cultured under ULA conditions for 0, 4, 7, and 14 days. Analyzed by two-way ANOVA and Sidak’s multiple comparison test. Data are presented as mean ± SEM. *n* = 4. Significantly different as indicated, * *p* < 0.05; *** *p* < 0.001. (**B**) Representative overlay images showing cell membrane integrity, via propidium iodide (PI) staining, of cells and MTS in ULA culture for 0, 4, 7, and 14 days. *n* = 4. (**C**) hematoxylin and eosin (H&E) staining of MOE PTEN^shRNA^ MTS after seven days under ULA conditions. *n* = 4. Scale bar= 100 μm.

Cell junctions, which are important in maintaining cell viability and preventing anoikis during metastasis, have also been suggested as mediating MTS formation [16]. To quantify the viability of cells that form MTS as compared to a control cell line that does not, viability was measured in MOE SCR^shRNA^ and MOE PTEN^shRNA^ cells grown under ULA conditions. Viability at each day was normalized to viability on Day 0 to account for intrinsic differences between the viability of MOE SCR^shRNA^ and MOE PTEN^shRNA^ cells. By Day 4, the relative viability of MOE SCR^shRNA^ cells was only 2.5% of that of Day 0 and remained low (1.7–3.7% of that of Day 0) throughout the experiment. By contrast, at Day 4 the viability of MOE PTEN^shRNA^ cells was 28% of that of Day 0 and was significantly higher than the relative viability of MOE SCR^shRNA^ (*p* < 0.001). At Day 7, the viability of MOE PTEN^shRNA^ cells was 17% of that of Day 0, which was 4.6-fold higher than the relative viability of MOE SCR^shRNA^ (*p* < 0.05). After 14 days under ULA, the relative viability of both cell lines was approximately 1.5% of Day 0 values (Figure 4A).

To investigate the integrity of the cell membrane in ULA culture, propidium iodide (PI) was added to wells of MOE SCR^shRNA^ and PTEN^shRNA^ cells after 0, 4, 7, and 14 days under ULA conditions and imaged 15 min afterwards. On Day 0, only a few cells from both cell lines took up PI, indicating that the membrane of most cells was intact. By Day 4, many MOE SCR^shRNA^ cells were clearly PI positive. At Day 7 and 14, the majority of the remaining MOE SCR^shRNA^ cells were positive for PI. In MOE PTEN^shRNA^ cells, by Day 4 and 7 MTS were present, with only a few PI-positive cells (Figure 4B). Interestingly, many of the cells that did take up PI were also not part of the MTS, supporting the idea that cell-to-cell contact is responsible for increasing cell survival under ULA conditions. After 14 days under ULA conditions, most cells, regardless of type, stained positive with PI (Figure 4B). Healthy cells on the outside of the MTS might be able to prevent PI from reaching dying cells on the inside of the MTS. Therefore, to determine if cells in the center of the MTS were also healthy after 7 days, MOE PTEN^shRNA^ MTS were collected and subjected to hematoxylin and eosin (H&E) staining. The center of the MTS appeared healthy and contained clear nuclei and no signs of cell death (Figure 4C).

We also tested the integrity of the cell membrane in our fallopian tube budding model of MTS formation. PI was added to MOE SCR^shRNA^ and MOE PTEN^shRNA^ cells after 15 days of overgrowth in traditional 24-well cell culture plates. Most individual MOE SCR^shRNA^ and MOE PTEN^shRNA^ cells floating in the media took up PI, which is indicative of a leaking plasma membrane. By contrast, the MTS-like structures formed in the MOE PTEN^shRNA^ cells were able to exclude PI, indicating an intact cell membrane (Figure S2C).

### 2.4. MTS Attached to Ovarian Stroma Exposed during Ovulation

**Figure 5:**
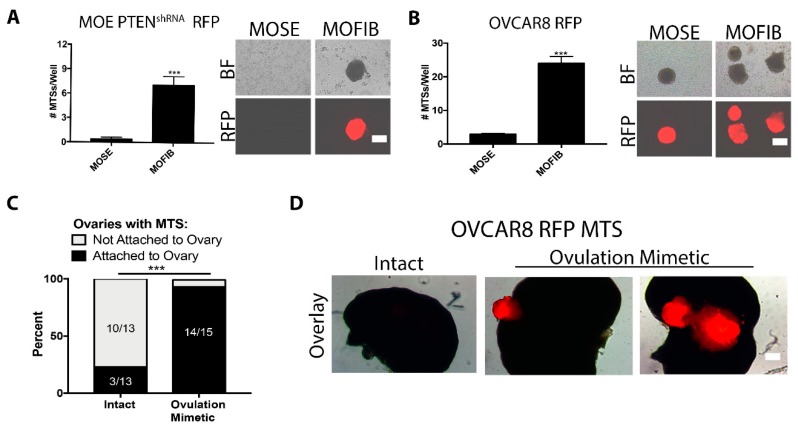
MTS attach to ovarian stroma exposed during ovulation. (**A**,**B**) Number of MOE PTEN^shRNA^ RFP (**a**) and OVCAR8 RFP (**B**) MTS attached to confluent monolayers of murine ovarian surface epithelia (MOSE) and murine ovarian fibroblasts (MOFIB) cells after 24 h with representative images. Data are presented as mean ± SEM and were analyzed by Student’s *t*-test. *** *p* < 0.001 relative to MOSE. *n* = 4. Scale bar = 10 μm. (**C**) Attachment of OVCAR8 RFP MTS to intact and ovulation mimetic ovaries. Analyzed by Fisher’s exact test. *** *p* < 0.001 relative to intact. *n* = 13–15. (**D**) Representative images of OVCAR8 RFP MTS attached to ovulation mimetic ovaries. Legend: BF, brightfield. Scale bar = 10 μm.

Ovulation is linked to the increased risk of developing ovarian cancer. Current evidence supports that the ovary is involved in primary metastasis of fallopian-tube-derived HGSOC due to exposure of ECM proteins during ovulation [12,14]. To test the role of the ECM, we measured the attachment of MOE PTEN^shRNA^ RFP and OVCAR8 RFP MTS to monolayers of murine ovarian surface epithelia (MOSE) cells or murine ovarian fibroblasts (MOFIB) cells to mimic the intact and ovulated ovary, respectively. MTS from both cell lines attached to MOFIB more than MOSE cells (31- and 8-fold, respectively; *p* < 0.01, Figure 5A,B). We analyzed the ability of OVCAR8 RFP MTS to attach to intact or ovulation mimetic ovaries using our previously established assay, in which ovaries are wounded artificially to mimic the exposure of the internal components of the ovary after ovulation in a hormone-independent manner [9,14]. After 24 h of incubation, 93% of ovarian mimetic ovaries had MTS attached. Interestingly, MTS attached at the wounded site. By contrast, only 6% of intact ovaries had an MTS attached (Figure 5C,D; *p* < 0.001). These results suggest that MTS from the fallopian tube may attach to the ovary at the site of ovulation or after menopause when some of the OSE is lost [26].

**Figure 6:**
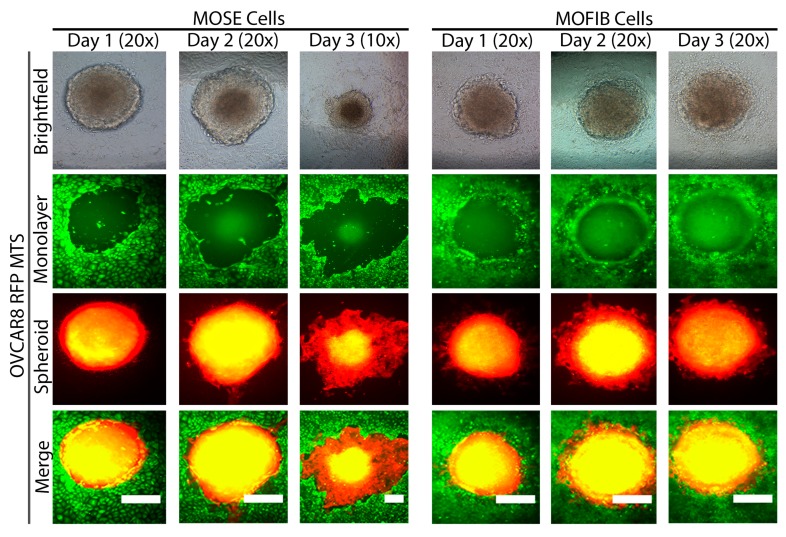
Representative images of the area cleared by OVCAR8 RFP MTS when plated on MOSE (left) or MOFIB (right) cells over three days. The area of MOSE cells cleared by OVAR8 RFP MTS was enlarged on Day 3, but the area of MOFIB cells cleared over three days remained constant. *n* = 4. Scale bar = 25 μm.

To determine if the RFP-tagged MTS were able to displace monolayers of MOSE or MOFIB cells, both monolayers were labeled with green CellTracker dye. On Day 1, OVCAR8 RFP MTS had displaced approximately 0.1 mm^2^ in both the underlying MOSE and MOFIB cells. The area of MOFIB cells remained constant over the next two days. With the MOSE cells, the area displaced by OVCAR8 RFP MTS remained constant on Day 2, but by Day 3, the area had increased approximately 3-fold to 0.4 mm^2^ and was dramatically larger than the area displaced on MOFIB cells (Figure 6 and Figure S3A; *p* < 0.001). In some cases, the OVCAR8 RFP MTS plated on top of the MOFIB cells remained very localized, with very little spreading. However, some OVCAR8 RFP MTS spread out over the three days but spread over the top of the MOFIB cells without displacing them (Figure S3B). These results indicate that MTS structures can displace both OSE and fibroblasts during initial attachment, but that fibroblasts are more resistant to displacement than the OSE as the MTS spread.

### 2.5. MTS Form Tumors after Intrabursal Xenograft

**Figure 7:**
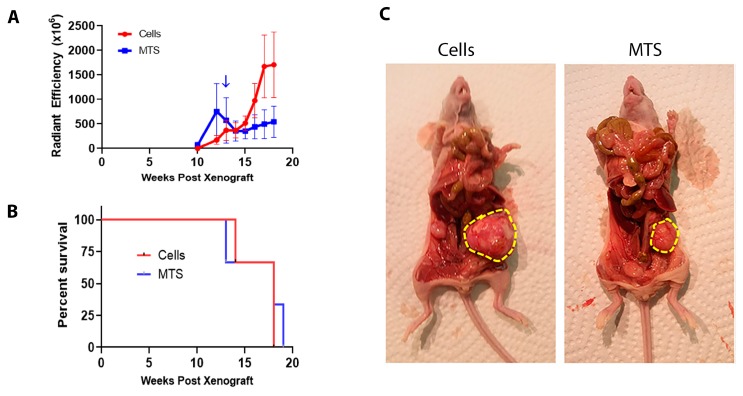
OVCAR8 RFP cells grown on traditional cell culture plastic (cells) or as MTS prior to being xenografted into the ovarian bursa are equally aggressive. (**A**) Average radiant efficiency ([p/s/cm^2^]/[μW/cm^2^]) ± SEM in mice with OVCAR8 cells tagged with RFP and xenografted as loose cells or MTS into the left ovarian bursa. ↓ represents when the mouse was sacrificed at a humane endpoint. Analyzed by two-way ANOVA. No significant differences were detected. *n* = 3. (**B**) Kaplan-Meier survival curves for mice in both treatment groups. Analyzed by log-rank (Mantel-Cox) test. No significant differences were detected. *n* = 3. (**C**) Representative images showing large ovarian tumors (circled in yellow) found in both treatment groups.

Next, we aimed to determine if MTS could colonize the ovary in vivo and to compare the tumorigenicity of loose cells and MTS. To this end, we xenografted a total of 38 MTS (2000 cells per MTS) formed with OVCAR8 RFP cells or an equal number of OVCAR8 RFP cells (76,000) grown on traditional cell culture plastic into the left ovarian bursa of athymic nude mice. Confirming that OVCAR8 RFP MTS could colonize the ovary in vivo, these mice developed tumors. One OVCAR8 RFP MTS mouse developed tumors quickly and required sacrifice at Week 14. The other two mice required sacrifice by Week 19. Contrary to our expectations, mice xenografted with OVCAR8 RFP cells developed tumors in a very similar time frame, requiring sacrifice at Week 19 due to ascites (Figure 7A,B). In both treatment groups, very large ovarian tumors were observed in the xenografted ovary (left) with smaller tumors spread throughout the peritoneum (Figure 7C). These results indicate that cells and MTS are approximately equal in their ability to form ovarian tumors in vivo.

## 3. Discussion

**Figure 8:**
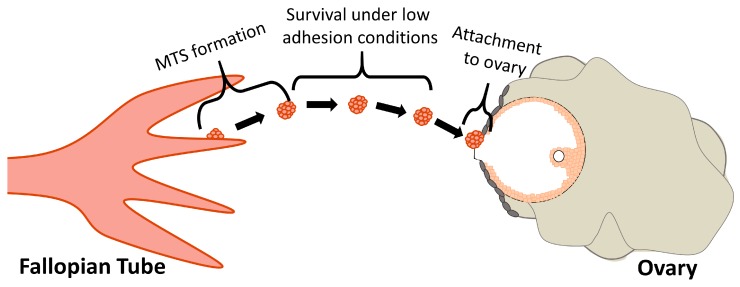
Schematic outlining the three steps necessary for MTS to mediate metastasis from the fallopian tube to the ovary. (1) MTS formation. After genetic alterations (e.g., loss of *PTEN*), the FTE overgrow and collectively detach from the fimbria. (2) Survival under low adhesion conditions. Cell-to-cell contacts in the MTS enhance cell survival while the cells are traversing from the fallopian tube to the ovary. (3) Attachment to the ovary. MTS preferentially attach at the site of ovulation due to the loss of OSE. However, if the MTS attach elsewhere, the MTS can displace OSE cells to invade the ovary.

Previous evidence suggests that colonization of the ovary is an important step in metastases of FTE-derived tumors to the peritoneum [8,14,27]. To date, most previous research has studied the role of individual cells as they exfoliate from STICs in the FTE and colonize the ovary. By contrast, the current study suggests that MTS may mediate metastasis from the fallopian tube to the ovary, which can be broken down into three major steps (Figure 8). First, reactive oxygen species released during ovulation cause genetic damage (e.g., loss of *PTEN*) to the FTE [28,29]. Damage in the FTE results in STIC formation, overgrowth of the epithelia [30], and eventually collective detachment of MTS from the epithelial monolayer. Second, the MTS move from the fallopian tube fimbria to the ovary. During this time the cell-to-cell contacts within the MTS help prevent anoikis and maintain cell viability. Finally, the MTS attach to the ovary. While our data indicate that the preferred site of attachment is the ovulatory wound, our results also show that MTS can displace OSE cells if they attach elsewhere on the ovary. After attachment to the ovary, chemotactic factors primarily secreted by the granulosa cells (e.g., activin A and norepinephrine [11,13]) stimulate invasion into the ovary. Interestingly, previous work on peritoneal metastasis indicates that a single MTS can form a metastatic tumor [20].

Our finding that OVCAR8 cells and MTS were equally tumorigenic may indicate that cells metastasize from the FTE to the ovary in both formats. Several factors influence whether an ovarian tumor from the FTE originates as a single cell or an MTS. For example, the ratio of individual cells to MTS that exfoliate from the primary tumor, the number of individual cells and MTS that attach to the ovary, and the average number of cells in each MTS each affect the percentage of cases that originate from an MTS. A recent study of eight HGSOC patients found STICs in all eight, but identified MTS in only three patients [22], indicating that not every patient with a STIC also has MTS in the fallopian tube. Hence, is clearly too early to use MTS as a prognostic marker in ovarian cancer patients. Since individual cells and MTS both exfoliate from STICs, the presence of STIC is likely still the best prognostic marker for patient survival. More work is required to determine if ovarian tumors in HGSOC patients are the results of individual cells or MTS metastasized from the fallopian tube.

In cBioPortal, PTEN deletion in the genome occurs in less than 10% of cases, but PTEN loss may be more common than is appreciated. Allelic imbalances at the PTEN locus have been detected in 39% of ovarian cancer samples [31]. DNA sequencing of laser-captured microdissected STIC lesions has found that one copy of PTEN was lost in 4/9 patients and another patient had a somatic mutation in PTEN [3]. Two different miRNAs overexpressed in ovarian cancer that specifically target PTEN have been identified [32,33], and immunohistochemistry has revealed that PTEN protein is absent in roughly 33% of STICs [34]. Examining PTEN protein expression in HGSOC patients, Martins et al. [35] found that PTEN was low or absent in >50% of HGSOC cases. Collectively, it appears that PTEN expression is lost in a large number of HGSOC cases through a combination of DNA alterations and transcriptional and translational mechanisms.

The ability of cell lines to form MTS varies widely between a panel of human cancer cell lines and our panel of murine-derived models [23]. OVCAR8 formed spherical compact MTS while OVCAR4 cells did not, which agrees with Shelby et al. [23], who screened the entirety of NCI-60 cell lines to identify cell lines that formed MTS-like structures. To our knowledge, this is the first study to start with normal cells that do not form MTS and alter genes in a systematic way to identify key genes in MTS ovarian cancer formation. While PTEN is best known as a negative regulator of AKT, constitutively active AKT (AKT^myr^) was not sufficient to induce MTS formation, indicating PTEN^shRNA^ has additional effects beyond AKT activation. In agreement with this, we have shown that Rac1/Jnk and Wnt4 signaling are important in mediating colonization of the ovary by MOE PTEN^shRNA^ cells [9,14]. Future work should explore how the loss of PTEN leads to MTS formation.

Mutations in p53 occur in >96% of HGSOC tumors and stabilization of p53 is one of the earliest lesions present in the development of HGSOC [3,36]. In the current study we only tested p53^R273H^, but point mutations can occur throughout the p53 gene. Hence, it is possible that PTEN loss would interact differently with other p53 mutations. The frequency of PTEN protein loss and p53 mutation suggests that these alterations frequently co-occur in HGSOC. We have shown in two different models that loss of PTEN in the FTE results in tumors with stabilized p53 [7,9], clearly indicating that PTEN loss affects p53 stability and function. In agreement with this, loss of p53 function has been found to be a prerequisite for tumorigenesis following loss of both PTEN alleles in prostate cancer [37]. Clearly there is important interplay between p53 and PTEN that remains to be elucidated.

The ovary is unique in that the OSE, at the site of ovulation, is lost every month, and the process of ovulation is linked with ovarian cancer [38,39]. Our results show that MTS preferentially attach to monolayers of ovarian fibroblasts as compared to the OSE, suggesting that MTS may adhere to the site of ovulation. In agreement, Yang-Hartwich et al. [12] have found that mCherry-labeled ovarian cancer cells adhere to the site of ovulation. Dean et al. [14] have found that rupture of the ovarian surface increases attachment of MOE cells to the ovary. While both of these studies used single cell solutions, taken together with our data, these studies support the idea of preferential adherence to the site of ovulation. Secondary metastasis, from the ovary to the omentum, is much different in that the peritoneal cavity is covered in a layer of mesothelial cells that are not lost regularly. Myosin-generated force in the MTS is required to clear the mesothelium [19]. Differences in the forces necessary to clear epithelial layers in the peritoneal space may contribute to the ovary being a preferred site of metastasis for FTE-derived tumors and explain, in part, the link between ovulation and ovarian colonization [3,8,12,27].

The number of ovulations is clearly linked to a woman’s risk of developing ovarian cancer [40], and yet most cases of HGSOC are diagnosed after menopause. There seem to be two possibilities for this apparent paradox. Tumor cells from the FTE might colonize the ovary before menopause due to the effects of ovulation. Tumors then remain confined to the ovary until menopause, after which widespread metastasis leads to diagnoses. A second possibility is that STICs form in the FTE due to the reactive oxygen species released during ovulation [28,29] but remain in the FTE until after menopause. The postmenopausal ovary is thought to be largely devoid of OSE [26]. Thus, our and others’ findings showing that cells and MTS attach to ovulation mimetic ovaries and the ovarian stroma [9,12,14] might indicate that it is easier for tumor cells and MTS to attach to the ovary after menopause. Further research is needed to evaluate these possibilities.

## 4. Material and Methods

### 4.1. Cell Lines and Cell Culture

MOSE and MOE (equivalent to human FTE) cells were generated from FVB mice and were originally donated by Dr. Barbara Vanderhyden, University of Ottawa. MOE cells stably expressing an empty plasmid (NEO) or plasmids encoding a scrambled shRNA (SCR^shRNA^), p53^R273^, KRAS^G12V^, AKT^myr^, PTEN^shRNA^, PTEN^shRNA^ + p53^R273H^, and PTEN^shRNA^ + KRAS^G12V^ had been previously generated by our lab and validated [7,24] and had recently been validated again by Western blot [14]. MOFIB cells were made by removing the OSE from the ovary of an FVB mouse using collagenase. The ovary was then further digested by incubation with trypsin and collagenase with frequent pipetting and vortexing. Cells were seeded in a 6-well plate and transfected with SV40. Cells were then passed to 10 cm dishes and incubated until colonies formed. Eight colonies were analyzed by Western blot. Clone 2 was used in subsequent experiments due to high vimentin expression and lack of cytokeratin 8 (Figure S4A). Clone 2 also had spindle-like morphology when subconfluent, but adopted a more cobblestone appearance at confluence (Figure S4B). The human FTE cell line FT33-TAg was a generous gift from Dr. Ronny Drapkin, University of Pennsylvania, PA. FT33-TAg cells were generated and cultured as described previously [41]. OVCAR4 (from NCI), OVCAR3 and OVCAR8 (both from American Type Culture Collection), and ES2 (donated by Dr. Maria Barbolina, University of Illinois at Chicago) had been validated by Short Tandem Repeat analysis within the previous two years. MOE, MOSE, OVCAR3, OVCAR4, and OVCAR8 cells were maintained in media as has been previously described [11,42,43]. MOFIB cells were maintained in MOE media. ES2 cells were maintained in RPMI 1640 media with 10% fetal bovine serum and 1× penicillin-streptomycin. All cultures were maintained in a humidified incubator at 37 °C and passed at confluence.

### 4.2. MTS Formation

Cells were collected with trypsin/EDTA, collected with media, counted, and diluted to 5000 cells/mL. Five hundred cells were added to each well of a 96-well, round bottom set of ULA plates (07-201-680, Corning, NY, USA). Cells were incubated for seven days. The contents of each well were imaged (undisturbed), pipetted up and down with a 200 μL pipette and imaged again (disturbed) with 4× or 10× objectives. To confirm MTS formation in a different model, the ability of MTS to form via budding from the monolayer was accessed as previously described [25]. Cells (25,000) were plated in 24-well plates and allowed to grow without passage for 15 days. MTS budding from the underlying monolayer was imaged at 4× and 20× on Days 1, 3, 5, 10, and 15. The number of MTS per well was counted and the diameters of the MTS were measured via ImageJ (National Institute of Health, Washington DC, USA) using 20× images. Experiments were performed on 2–4 wells on each plate. Experiments were replicated on at least four separate plates.

### 4.3. Viability Assay

To measure the viability of cells and MTS in ULA culture, 100 μL of CellTiter-Glo 3D Reagent (G9681, Promega, Madison, WI, USA) was added to each well and incubated for 15 min. The contents of each well were transferred to a white 96-well plate (07-200-761, Corning) and luminesce was measured on a Synergy HT BioTek plate reader. To identify which cells in 3D culture had developed a leaky cell membrane (an indicator of late-stage apoptosis), 20 μg/mL of propidium iodide (P4170, Sigma Aldrich, St. Louis, MO, USA) was added to each well and incubated for 15 min. At least three technical replicates were present on each plate. Experiments were replicated on four independent plates. Cells and MTS were imaged with an inverted Nikon TS-100 fluorescent microscope (Nikon, Melville, NY, USA).

### 4.4. MOSE and MOFIB Attachment Assay

To quantify attachment of MTS to confluent monolayers of MOSE or MOFIB cells, 36 fluorescently-labeled MTS were collected from ULA plates, centrifuged, resuspended in 500 μL of media, and added to one well of a 24-well plate. Plates were incubated for 24 h, decanted, and washed, and the number of MTS attached to the monolayer were counted. Experiments were performed on 2–4 wells on each plate. Experiments were replicated on at least on separate plates.

### 4.5. MTS Clearance Assay

The ability of MTS to clear MOFIB and MOSE cells was measured based on a similar assay to measure the clearance of mesothelial cells [44]. MOFIB (2000) or MOSE (100,000) cells per well were plated on 24-well plates and allowed to grow until confluent (over about four days). The monolayers were labeled with green CellTracker dye (C7025, Invitrogen, Waltham, MA, USA) following the manufacturer’s instructions. MTS (3–12 per well) were added to each well and allowed to attach overnight. Every day for the next three days the media in each well was replaced and attached MTS were imaged on a Nikon TC200 microscope with brightfield and fluorescence imaging. Green and red fluorescence images were merged and the area displaced by the MTS in the green fluorescent images was quantified using ImageJ. Experiments were performed on three wells on each plate. Experiments were replicated on at least four separate plates.

### 4.6. Hematoxylin and Eosin Staining of MTS

After 7 days under ULA conditions MOE PTEN^shRNA^ MTS were collected and embedded in alginate beads [45]. The beads were fixed in 4% paraformaldehyde, dehydrated, and embedded in paraffin. Sections (5 μm) were deparaffinized, stained with H&E, and mounted. Images were captured at 40× using a Nikon Eclipse E600 microscope (Nikon, Melville, NY, USA).

### 4.7. Mouse Studies

All animals were treated in accordance with the National Institutes of Health (NIH) Guidelines for the Care and Use of Laboratory Animals and the established Institutional Animal Use and Care (IACUC) protocol at the University of Illinois at Chicago (protocol numbers: 17-174 and 17-150).

### 4.8. Ex Vivo Ovarian Attachment Assay

Attachment of MTS to ex vivo murine ovaries was assessed using our recently described attachment assay [9,14]. Ovaries were removed from CD-1 mice, age 16–18 days, which were obtained from in house breeding. Ovaries were dissected free of the reproductive tract and bursa. Ovaries were left intact or the ovarian surface was ruptured with a scalpel blade to expose the ECM (ovulation mimetic). Ovaries were incubated with 12 OVCAR8 RFP MTS for 24 h on an orbital shaker at 37 °C. The next day ovaries with MTS attached were counted. As the hilus represents a “wound” on both the intact and ovulation mimetic ovaries, MTS attached to this region were not included in the analysis. Representative RFP and brightfield images were captured and an overlay image was produced by copying the red pixels from the RFP images and pasting them on top of the brightfield image using ImageJ.

### 4.9. Transgenic Mice and Immunohistochemistry

C57b/6 LoxP-PTEN-LoxP were obtained from the Mouse Models of Human Cancer Consortium and bred with mice expressing CRE-recombinase under the control of a PAX8 promoter from the Research Institute of Molecular Pathology, Vienna [46]. Tissue-specific expression of CRE and loss of PTEN has been previously validated [9]. Reproductive tracts were embedded in paraffin and prepared for immunohistochemistry (IHC) as described previously [7]. Tissues were incubated with primary antibodies overnight (Table S1). Images were acquired on a Nikon Eclipse E600 microscope using a DS-Ri1 digital camera and NIS Elements software (Nikon, Melville, NY, USA,).

### 4.10. Xenograft Study

OVCAR8 RFP cells were either grown on traditional cell culture plastic or grown as MTS as described above for seven days. Cells (76,000) or MTS (38 MTS; 2000 cells per MTS) were collected, moved into phosphate buffered saline, and injected into the left ovarian bursa of 8-week old athymic nu/nu mice (Taconic Germantown, NY, USA). Tumor growth was imaged every two weeks starting at four weeks and every week starting at Week 12. Imaging was done with a Xenogen IVIS Spectrum In Vivo Imaging System (PerkinElmer, Waltham, MA, USA) as previously described [47,48].

### 4.11. Statistical Analysis

All data have been presented as mean ± SEM. Continuous data with two treatment groups were analyzed with a Student’s *t*-test. Categorical data were analyzed by Fisher’s exact test. Experiments with continuous data and only two treatments were compared using Student’s *t*-test. Experiments comparing two treatments over time were analyzed by two-way ANOVA with Sidak’s multiple comparisons test comparing treatments (e.g., cell lines) within the same time point. All analyses were performed in Prism 7.0a. *p* < 0.05 was considered statistically significant.

## 5. Conclusions

In conclusion, our data indicate that the loss of PTEN in the FTE is sufficient to drive MTS formation. The ability of cells to form MTS under ULA conditions increases the survival of FTE cells which gives the cells more time to attach to the ovary after exfoliating from the FTE. MTS seem to attach to the ECM which is exposed after the loss of the OSE. However, loose cells and MTS were equally aggressive in mice. These results suggest that MTS-like structures may be important mediators of metastasis from the fallopian tube to the ovary.

## Supplementary Materials

The following are available online at https://www.mdpi.com/2072-6694/11/6/884/s1, Figure S1: (A,B) The diameter of MTS formed by OVCAR8 and ES2 (A) or MOE cells expressing PTENshRNA, p53R273H, and KRASG12V (B) after seven days in ULA. (C) Representative images of MOE NEO and MOE SCRshRNA cells after seven days in ULA culture, Figure S2: (A) Number of MTS-like structures per well after growth of MOE SCRshRNA and MOE PTENshRNA cells. Diameter of MTS structures formed by overgrowth of MOE SCRshRNA and MOE PTENshRNA cells. Representative images of MOE SCRshRNA and MOE PTENshRNA cells overgrown in a 24-well plate for 15 days and treated with PI for 15 minutes. MTS present in BF image of MOE PTENshRNA cells, is also present in fluorescent image but not visible, Figure S3: (A) Area cleared by OVCAR8 RFP MTS over three days when plated on MOSE and MOFIB cells. (B) Representative images showing that by day 3, some OVCAR8 spheroids on monolayers of MOFIB cells remain localized, with very little spreading or migration, over three days (top), Figure S4: (A) Representative western blot for vimentin and cytokeratin 8 in eight MOFIB clones. (B) Morphology of MOFIB2 cells before and after reaching confluence. Table S1. Antibodies and conditions used for immunohistochemistry (IHC) and western blots (WB) to detect PAX8, p53, vimentin (VIM), cytokeratin 8 (KRT8), and β actin.
