# Supplementary material for: Loss of PTEN in Fallopian Tube Epithelium Results in Multicellular Tumor Spheroid Formation and Metastasis to the Ovary
Source: Cancers (Basel). 2019 Jun 25;11(6):884. doi: 10.3390/cancers11060884 (PMC6627669; doi:10.3390/cancers11060884)
Supplement: Supplementary file 1 [file cancers-11-00884-s001.pdf]

Supplementary Materials: Loss of PTEN in Fallopian Tube Epithelium Results in Multicellular Tumor Spheroid Formation and Metastasis to the Ovary

Matthew Dean, Vivian Jin, Tova M. Bergsten, Julia R. Austin, Daniel D. Lantvit, Angela Russo and Joanna E. Burdette

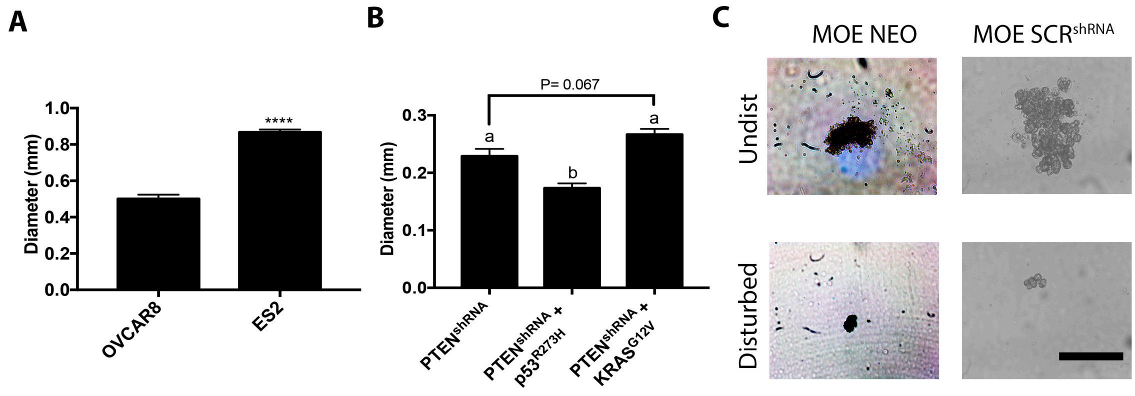

Figure S1. (**A**,**B**) The diameter of MTS formed by OVCAR8 and ES2 (**A**) or MOE cells expressing PTEN^{shRNA}, p53^{R273H}, and KRAS^{G12V} (**B**) after seven days in ULA. (**C**) Representative images of MOE NEO and MOE SCR^{shRNA} cells after seven days in ULA culture. Scale bar: 250 µm. Mean ± SEM. $n = 4$. **** Indicates $p < 0.0001$ relative to OVCAR8.

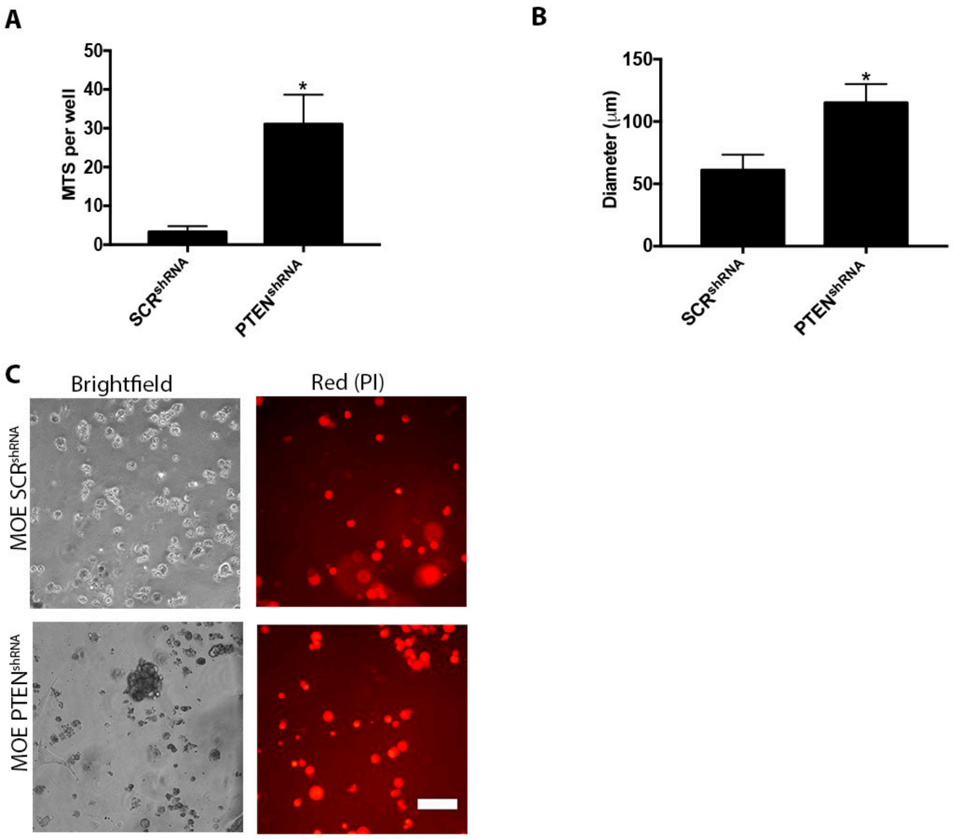

Figure S2. (**A**) Number of MTS-like structures per well after growth of MOE SCR^{shRNA} and MOE PTEN^{shRNA} cells. (**B**) Diameter of MTS structures formed by overgrowth of MOE SCR^{shRNA} and MOE PTEN^{shRNA} cells. (**C**) Representative images of MOE SCR^{shRNA} and MOE PTEN^{shRNA} cells overgrown in

a 24-well plate for 15 days and treated with propidium iodide (PI) for 15 minutes. MTS present in brightfield image of MOE PTEN^{shRNA} cells, is also present in fluorescent image but did not take up PI. Scale bar = 20 µm. mean ± SEM. n = 4. Significantly different * $p < 0.05$.

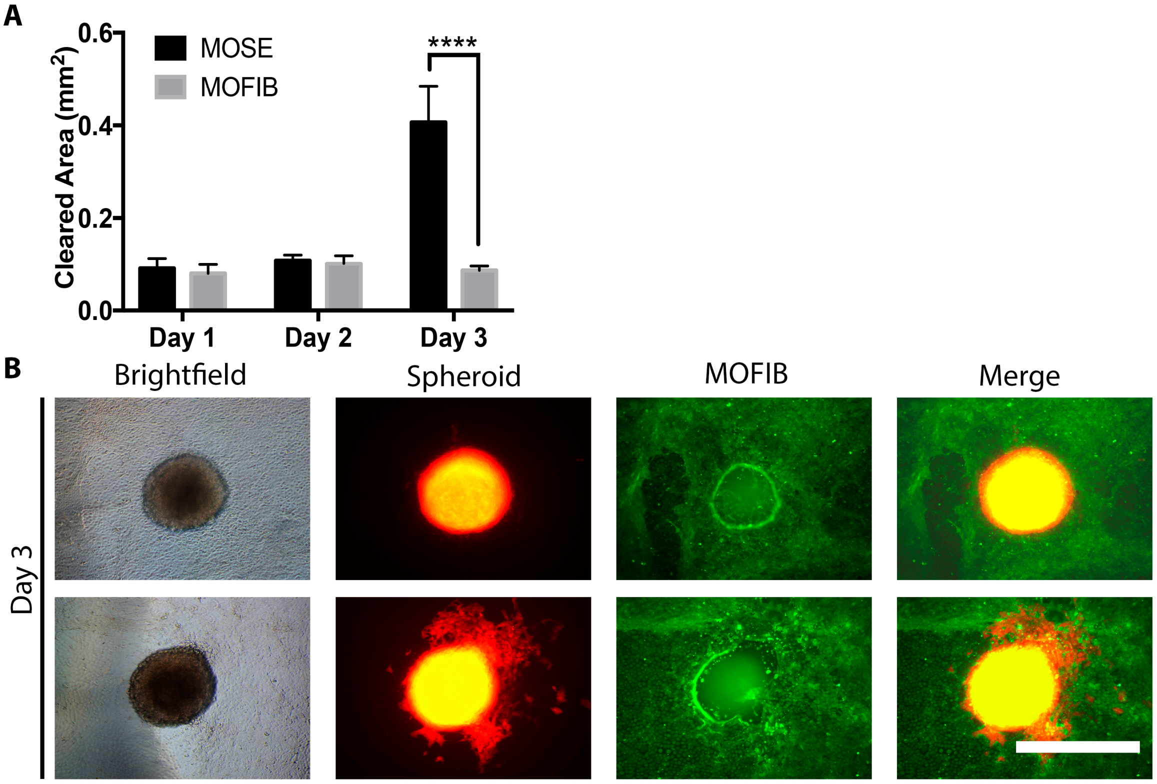

Figure S3. (**A**) Area cleared by OVCAR8 RFP MTS over three days when plated on MOSE and MOFIB cells. (**B**) Representative images showing that by day 3, some OVCAR8 spheroids on monolayers of MOFIB cells remain localized, with very little spreading or migration, over three days (top). Other OVCAR8 RFP spheroids spread over the MOFIB cells, without displacing the underlying fibroblast cells (bottom). Mean ± SEM. n = 4. Scale bar: 250 µm.

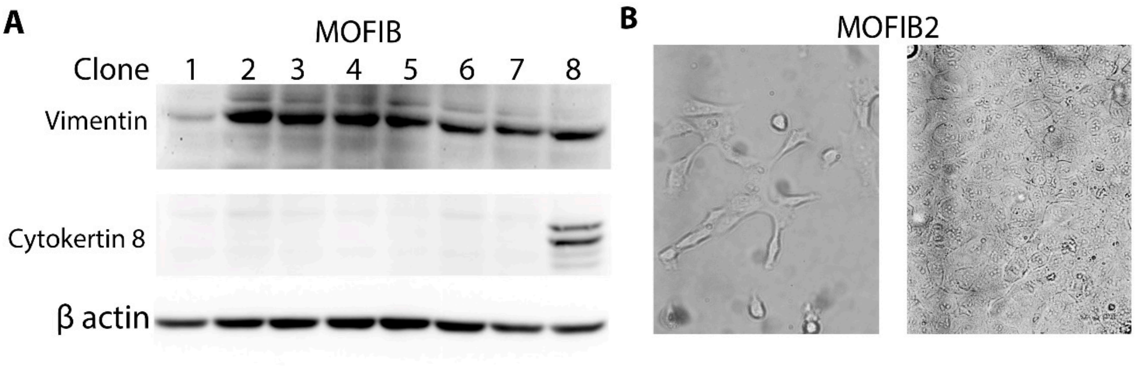

Figure S4. (**A**) Representative western blot for vimentin and cytokeratin 8 in eight MOFIB clones. (**B**) Morphology of MOFIB2 cells before and after reaching confluence.

Table S1. Antibodies and conditions used for immunohistochemistry (IHC) and western blots (WB) to detect PAX8, p53, vimentin (VIM), cytokeratin 8 (KRT8), and β actin. Antibodies were obtained from Protein Tech (Rosemont, IL, USA), Santa Cruz Biotechnology (Dallas, TX, USA), Cell Signaling (Beverly, MA, USA), Development Studies Hybridoma Bank (DSHB, University of Iowa, Iowa City, IA, USA), or Abclonal (Woburn, MA, USA).

Protein	Technique	Dilution	Blocking	Item Number, Company
PAX8	IHC	1:250	Serum/BSA	10336-1-1AP, ProteinTech
p53	IHC	1:50	Serum/BSA	SC-6243, Santa Cruz Biotechnology
VIM	WB	1:1000	milk	5741T, Cell Signaling
KRT8	WB	1:200	BSA	AB_531826, DSHB
β actin	WB	1:1000	milk	AC006, Abclonal

© 2019 by the authors. Licensee MDPI, Basel, Switzerland. This article is an open access article distributed under the terms and conditions of the Creative Commons Attribution (CC BY) license (http://creativecommons.org/licenses/by/4.0/).
